# Understanding disappearances in Mexico City: A data-driven analysis

**DOI:** 10.1371/journal.pone.0323405

**Published:** 2025-09-23

**Authors:** Daniel Aguilar-Velázquez, Ruben Calvario Peréz, Carlos Mondragón Mendoza, Alfonso Valenzuela-Aguilera

**Affiliations:** 1 Instituto de Física, Universidad Nacional Autónoma de México, Ciudad de México, México; 2 Escuela Superior de Cómputo, Instituto Politécnico Nacional, Ciudad de México, México; 3 Facultad de Arquitectura, Universidad Autónoma del Estado de Morelos, Cuernavaca, México; Facultad Latinoamericana de Ciencias Sociales Mexico, MEXICO

## Abstract

Mexico is facing an escalating crisis of violence, marked by a sharp increase in disappearances. However, key information remains unknown, such as the typical profile of victims, geographic hotspots, and the relationship between disappearances, economic conditions, and public security. We used a government database of 3,450 disappearances in Mexico City, together with scraped data, to analyze the phenomenon of missing persons. We found that disappearances are not homogeneously distributed along Mexico City, the central district has the highest disappearance rate per capita, which can be attributed to city mobility for job locations. Men account for 62.5% of missing persons, but women aged 15-19 are the most vulnerable group. There is a strong correlation (*r*=0.95) between reports of drug dealing and disappearances, both of which may be related to the presence of organized crime. Furthermore, when disappearances are normalized to account for mobility related to job locations, a strong negative correlation (*r*=–0.7) emerges between disappearances and housing prices. This suggests a pattern of socio-economic segregation in disappearances, with higher rates in areas with lower housing prices. Integrating data on disappearances, housing prices, reports of drug dealing, and perception of insecurity for each municipality, we implemented K-means algorithm. Without spatial information, K-means divided Mexico City in west and east. In the east side, people are more vulnerable to disappearances than those in the west side.

## Introduction

Mexico faces profound institutional challenges, particularly in the realm of justice and public security. The state often fails to ensure accountability and protect citizens’ rights, with widespread impunity that undermines trust in legal institutions [1,2]. Currently, more than 110,000 people are missing in Mexico [3], and since 2006 when the president Felipe Calderon launched the “war on drug cartels”, the number of homicides and disappearances has increased [1,4].

Around 90% of disappearances in Mexico have occurred since 2006, showing a persistent upward trend [5]. The 2018–2024 presidential term was the most violent, with 194,000 homicides and 60,000 disappearances [6], including 13,000 in 2024 alone [5]. Most cases are concentrated in areas controlled by organized crime [1], which uses disappearances for forced recruitment of men [7] and the trafficking of women [1], positioning organized crime as a key perpetrator.

Paradoxically, the war on drugs increased violence and disappearances. With this war, the government achieved the fragmentation of cartels, but new cartels were created from the fragments, became more violent and diversified the types of crime [8]. Evidence further suggests that the government avoided targeting the Sinaloa Cartel, facilitating its expansion and domain [9–11].

No single theory fully explains the disappearance phenomenon; it requires a several-theory approach [12,13], which also depends on the particular place. In this study, we analyzed disappearances in Mexico City because it is one of the main disappearance centers in Mexico [14]. Mexico City is characterized by a dense population (approximately 22 million people, including its metropolitan area), high socioeconomic diversity, socio-spatial segregation, and income inequality [15–18].

Three frameworks guide our analysis: criminogenic state [19], social disorganization and institutional anomie [20]. The criminogenic state describes a government that enables disappearances by omission or commission, reflected in a 98% impunity rate [21]. Social disorganization explains the concentration of crime in specific areas, often residential and gentrified [20,22], and is supported by Routine Activity and Crime Pattern Theories, which link crime with mobility: 7 million daily commutes in Mexico City [23–27]. Institutional anomie ties rising violence to weak rule of law and a profit-driven society, conditions evident in Mexico City [20].

Criminal violence is concentrated in the north and east of the City [20,28,29]. However, there is a lack of understanding as to whether disappearances are concentrated in specific areas of Mexico City. In this article, we explore the main characteristics of missing persons by analyzing the public version of the Mexican database of missing persons [30]. This database comprises 3,450 disappearances that occurred during 2018-2023. We aimed to identify the zones with the highest disappearance rates.

We explore whether living in an expensive zone involves a lower probability of being missed. Finally, for all municipalities, we integrate the columns involving income range, insecurity, and disappearances to elucidate the cluster of municipalities that need more help to reduce disappearances, inequality, and insecurity.

## Materials and methods

### Missing people database

We use the public version of the missing persons database internal registry (versión publica del registro interno de personas desaparecidas) [30]. This database was created in 2019 by the Mexico City Search Commission (Comisión de Búsqueda de Personas de la Ciudad de México, CBPCDMX). It contains records of people reported missing, comprising 3,450 entries of people who disappeared in Mexico City from 2019 to 2023. The database consists of 9 columns: Report date, sex, age group, nationality, missing date, municipality of disappearance, status (missing or located), location date, and geographic state of location (the last two only apply if the person was located). This database is available at [30] and does not indicate whether the persons located were found dead or alive.

### Normalized disappearances

To obtain a more precise measure of disappearances, we isolated disappearances from city mobility. In Mexico City and the metropolitan area, 7 million commutes are made every day [26,27], the majority of commutes made from the peripheral to central zones [27,31]. We propose a normalized version that is obtained dividing disappearances by the geometric mean between population and job offers.

$$normalized\;disappearances=\frac{disappearances}{\sqrt{population\times job\;offers}}$$ (1)

The geometric mean is useful when both quantities possess different scales (population in millions and job offers in thousands). For example, if we used the arithmetic mean (A+B)/2, the population would take an over-representation (more than 10 times higher than the job offers), and the job offers may not influence normalized disappearances. Instead, the square root of the geometric mean reduces the scale, avoiding extreme values. Job offers are used as a variable to quantify the mobility of citizens between municipalities. To obtain job offers for municipalities, we use the Mexican National Employment Service reported [32]. These offers correspond to formal jobs from private companies and government institutions. The total population per municipality is obtained from the National Institute of Statistics and Geography [33].

### Housing prices as a measure of income range

Given the high inequality observed in Mexico City, we tested the relationship between disappearances and socioeconomic status. To investigate this relationship, we obtained the average housing price per ${\text{m}}^{2}$ for each municipality. The price of housing has been used to identify the income range [34,35].

We performed web scraping to gather data on approximately 20,000 flat offers in Mexico City specifically for the year 2024. These offers included information on flat size in square meters, prices in Mexican pesos, and the corresponding neighborhood. The web-scraped data contain flats for sale (new and old) for all 16 municipalities in Mexico City. We obtained the average price per ${\text{m}}^{2}$ for each municipality. Bank auction flats were not taken into account as they are strongly associated with irregular sales below market prices [36]. We filtered duplicate flat offers and used the Python language with the BeautifulSoup library.

### Security variables: Perception of insecurity and reports of drug dealing

We also consider 2 additional variables: reports of drug dealing and perception of insecurity for each municipality. These two variables are introduced to measure the relation between disappearances and the general security environment. Reports of drug dealing are also normalized by the geometric mean of the population and job offers. This normalization is made because drug consumption depends on the total population.

$$normalized\;drug\;dealing=\frac{drug\;dealing}{\sqrt{population\times job\;offers}}$$ (2)

We obtained the number of reports of drug dealing for all municipalities using the Mexico City crime incidence database [37]. This database comprises preliminary investigations or investigation files of crimes reported by state and federal justice entities. Moreover, the perception of insecurity was obtained by National Institute of Statistics and Geography (INEGI), and represents the percentage of the population aged 18 years or older who resides in Mexico City who reported that living in their municipality was currently unsafe [38].

### Dimensional reduction

To obtain a more general sense of the risk zones, we applied dimensional reduction and clustering to identify general geographic patterns. We used Principal Component Analysis (PCA) to apply dimension reduction. Principal components, composed of linear combinations of variables, are obtained from the eigenvectors of the covariance matrix [39]. We obtained the covariance matrix from the variables: normalized disappearances, housing price per ${\text{m}}^{2}$, normalized reports of drug dealing, perception of insecurity, and located/disappearances.

Eigenvalues represent the variances of linear combinations [40], so one needs to find the highest eigenvalue, and then proceed to obtain the eigenvector associated with the eigenvalue. The elements of the eigenvectors are called PC loadings and represent the weights for each variable in the reduced dimension.

### K-means algorithm

We use the unsupervised machine learning algorithm K-means [41], which clusters unlabeled data into K different groups. Each municipality represents a point in the N-dimensional space, where N is the number of attributes. We used two attributes (N = 2), PC1 and PC2, obtained from the dimensional reduction. The algorithm consists of: (1) It is proposed K centroids in the N-dimensional space, (2) the Euclidian distance is computed between centroids and points, (3) Each point is assigned to the nearest centroid forming a cluster for each centroid, (4) for each cluster of points, it is computed its new mean (new centroid position), (5) the steps (2) to (4) continue iterated until no change in the assignements are registered.

All PC values are normalized to obtain values between 0 and 1, and then we apply the K-means algorithm. To determine the number of clusters K, we used the elbow method [42], which results in *K* = 2, in this case.

### Limitations of the study

The principal limitation of the work is the low spatial resolution of the missing people database. The database indicates the municipality of the disappearance for each register, and although we have 3,450 rows, only can be categorized in 16 municipalities. The correlations and patterns we found are limited to 16 areas of resolution. This spatial resolution has been applied in recent and peer-review studies to characterize crime and economics in Mexico City [20,22]. In addition, the database does not specify whether the people located are dead or alive. This represents a problem because we cannot infer whether the location of people is a positive or negative parameter.

## Results

### Disappearances by gender, age, and year

Fig 1A shows the probability distribution of disappeared persons by age, the most frequent value for women is observed at 15-19 years. For men, a wide distribution is shown with a less notorious maximum at 30-34 years. A $p<1\;\times \;10^{-4}$ value is found between both distributions in a two-tailed t-test, indicating a statistical difference, i.e., men disappeared older than women.

**Fig 1:**
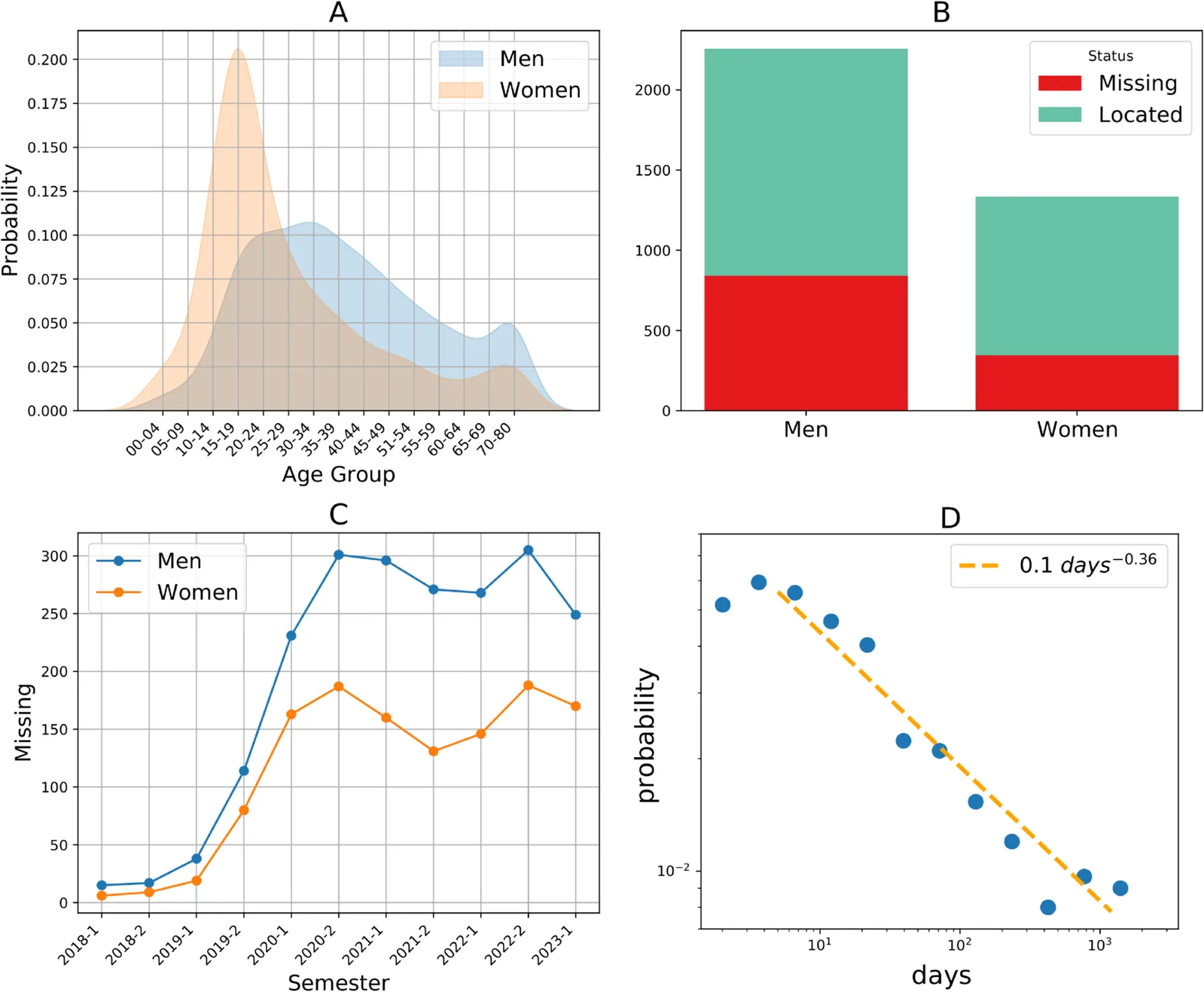
Characterization of missing people. A) Age probability distributions for missing people by gender. Female disappearances are characterized by a peak in the 15-19 year age group, whereas the age distribution for men corresponds to typical working life duration. A $p<1\;\times \;10^{-4}$ value indicates a statistical difference between the two distributions. B) The bar chart shows men and women who are still missing (green bar), located (red bar), and total disappearances (sum of still missing and located people). There are more male disappearances than female, and men have a lower probability of being located. C) Time series of missing people by gender. There were more missing people before and after the COVID-19 pandemic. D) Probability distribution of the number of days people were missing (only for located people). The distribution approximately follows a power-law behavior $P(days)\approx A\times days^{-\alpha }$, where α=0.36 and *A* = 0.1.

Fig 1B shows the total disappearances per gender, people located (green) and people that remain missing (red). We observe that there are more men disappearances (2,156) than woman disappearances (1,295). For men: missing men=781, located=1,375, and located/disappearances=63.77%. For women: missing women=321, located=974, and located/disappearances=75.21%. The values found here are relative to the total population considering that 52.2% of the population are women and 47.8% are men [33]. These results indicate that the probability for men to return home after disappearing is smaller than for women.

In Fig 1C we show a semester-based time series of missing people by gender, starting from 2018. There is a rapid increase beginning in that year, which decelerates in 2020, possibly linked to the COVID-19 pandemic. However, for the second semester of 2022, when the pandemic was considered over, missing people reached pre-pandemic levels.

Fig 1D shows the probability of the number of days located persons were missing. We found that the probability (*P*) approximates the behavior of the power law: P(days)≈A × $days^{-\alpha }$, where α=0.36 and *A* = 0.1. For the estimation of the power-law exponent, we used the ML* method [43], which is suitable for the estimation of power-law exponents that are located within the range 0<α≤1. We found α=0.36 ± 0.11, where ± indicates the mean square error of the power-law fit. We found no statistical differences between the distributions for men and women (p-value=0.17, Kolmogorov-Smirnov test). The distribution also indicates that the majority of people are located within the first 2 months. However, a tiny portion of people are located after 1,000 days (3 years).

### Geographic distribution of missing people

Fig 2A shows the number of disappeared people in Mexico City by municipality. We observe that the majority of disappearances occur in Iztapalapa (east of Mexico City), Cuauhtémoc (downtown), and Gustavo A. Madero municipalities (north of Mexico City). However, Iztapalapa and Gustavo A. Madero are densely populated zones, with 1.7 million and 1.1 million people, respectively [33]. To remove the effect of population size, we normalized the number of disappearances by dividing the number of inhabitants in each municipality. Fig 2B shows the number of disappearances per inhabitant, with the highest value found in Cuauhtémoc municipality. However, Gustavo A. Madero and Iztapalapa municipalities no longer feature prominently. Fig 2C displays the ratio of located people to disappearances. We observe a cluster of municipalities with a high percentage of located people, including Iztacalco, Tlalpan, Coyoacán, Benito Juárez, and Magdalena Contreras. The latter three municipalities are characterized by middle-class income levels.

**Fig 2:**
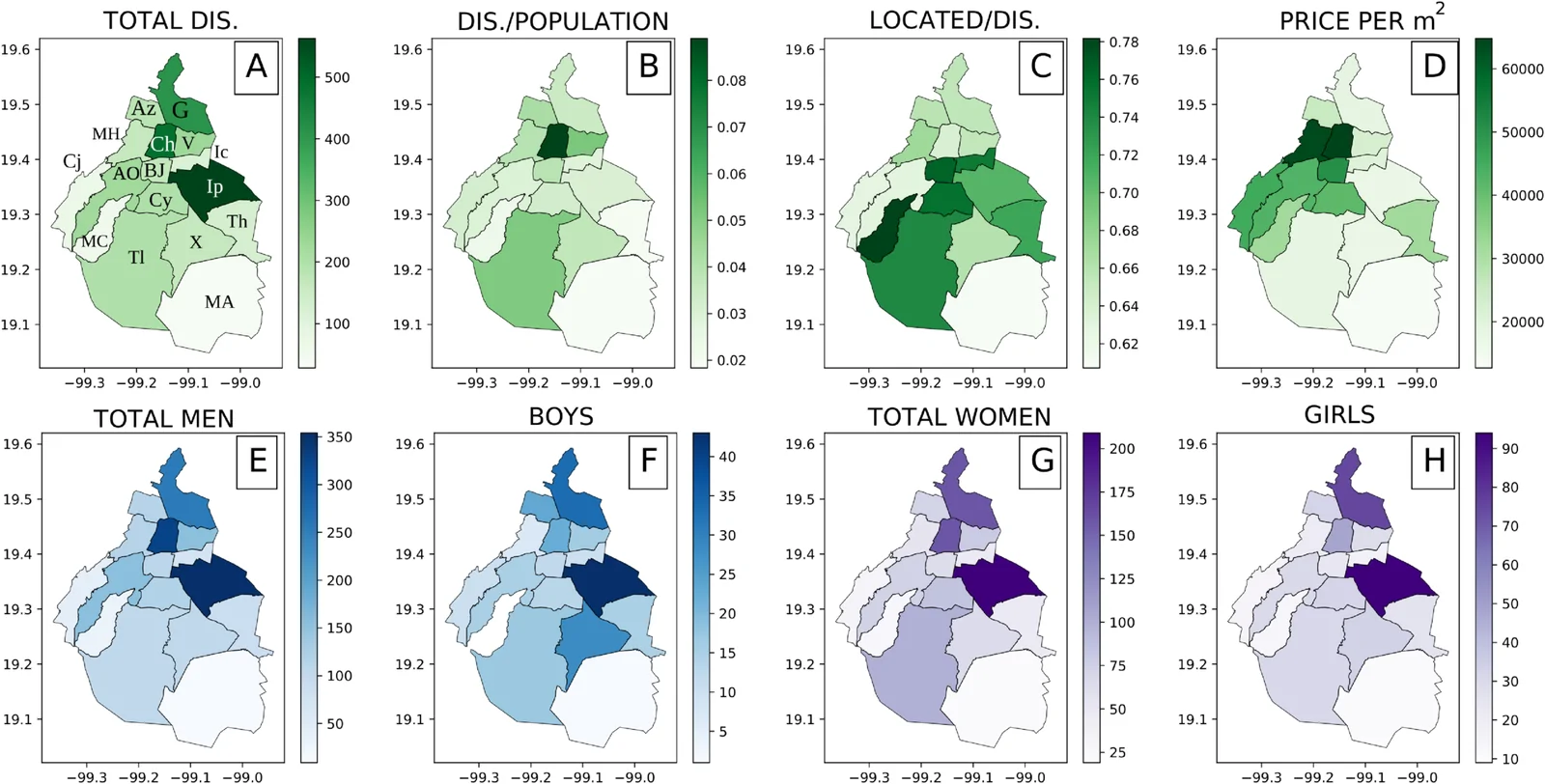
Maps of disappeared people in Mexico City. A) Disappeared people per municipality. B) Disappeared people divided by the population. C) Located people divided by disappeared people. D) Average housing price per ${\text{m}}^{2}$. E) Disappeared men. F) Disappeared Boys (younger than 18 years old). G) Disappeared women. H) Disappeared girls (younger than 18 years old). Municipality abbreviations: Alvaro Obregón (AO), Azcapotzalco (Az), Benito Juárez (BJ), Coyoacán (Cy), Cuajimalpa (Cj), Cuauhtémoc (Ch), Gustavo A. Madero (G), Iztacalco (Ic), Iztapalapa (Ip), La Magdalena Contreras (MC), Miguel Hidalgo (MH), Milpa Alta (MA), Tlahuac (Th), Tlalpan (Tl), Venustiano Carranza (V), and Xochimilco (X).

Using scraped data, Fig 2D shows the average housing price (in mexican pesos) per ${\text{m}}^{2}$ in each municipality. We include this variable to analyze whether expensive areas correlate with more security or fewer disappearances. We observe a cluster of high prices in the west-central zone of Mexico City. Cuauhtémoc (Ch) and Miguel Hidalgo municipalities (MH) are the most expensive areas, followed by Benito Juárez, Coyoacán, la Magdalena Contreras and Alvaro Obregón. Previous work indicates that Cuauhtémoc and Miguel Hidalgo municipalities contain gentrified and super-gentrified zones [18].

In Fig 2E, we show total adult men and boys disappearances, and only boy disappearances (Fig 2F). The municipality with the most boy disappearances is Iztapalapa. However, for adult men, the most dangerous municipalities are Cuauhtémoc and Iztapalapa. In Fig 2G we show total adult women and girls disappearances, and only girl disappearances (Fig 2H). Similar to boys, the most dangerous municipality for girls is Iztapalapa, and for adult women, it is Cuauhtémoc and Iztapalapa. These results may be attributed to the fact that Iztapalapa is the municipality with the most inhabitants and that many adults travel to downtown Cuauhtémoc for work.

### Normalized disappearances and its relationship with the economic and security environment

To isolate disappearances from the population size and mobility, we propose a normalized measure of disappearances. The disappearances of each municipality is divided by the geometric mean between population and job offers (see Materials and methods). Fig 3A shows the map for normalized disappearances. The east side shows the highest disappearance rates per normalized exposure-that is, after adjusting for both size and commute density. G, Th, and X are the municipalities with the highest normalized disappearances, and BJ shows the lowest value. The normalized disappearance for Th is 3.4 times higher than for BJ.

**Fig 3:**
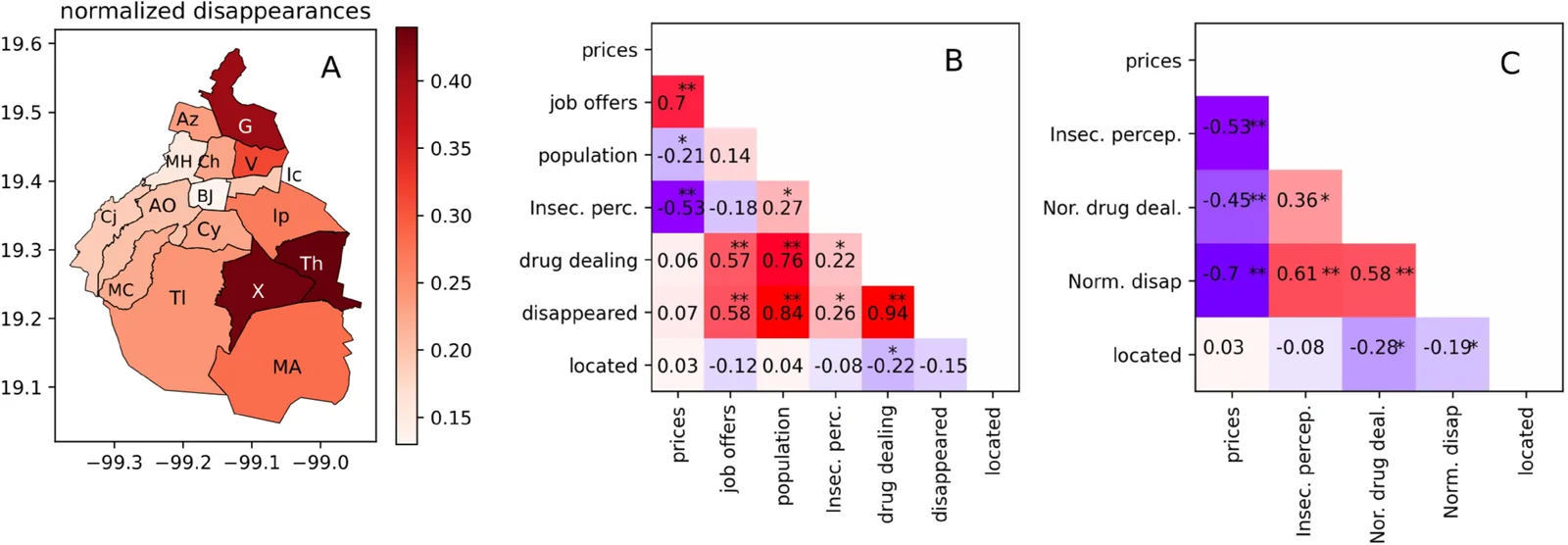
Normalized disappearances and Pearson correlation coefficients. A) Map of normalized disappearances. The east side of the city exhibits the highest rates of disappearances. B) Pearson correlation coefficients considering raw variables. Disappeared people is strongly correlated with reports of drug dealing, both variables also are correlated with the population size and job offers (citizens mobility). C) Normalized disappearances and normalized reports of drug dealing are considered. Housing prices are negatively correlated with disappearances, reports of drug dealing and perception of insecurity. * indicates *p* – *value* < 0.05, and ** indicates *p* – *value* < 0.01.

Then, to analyze the relationship between disappearances and the economic and security environment, we compute the Pearson correlation coefficient. First, we probe the raw variables (without normalization). In Fig 3B we show Pearson’s correlation matrix between the raw variables: housing prices, job offers, population, perception of insecurity, drug deals reports, disappearances, and located individuals.

Strong correlations are found among disappearances, job offers, population, and reports of drug dealing. The strongest correlation is found between reports of drug dealing and disappearances (*r* = 0.95). These results indicate that drug dealing and disappearances are mediated by the number of people in terms of population and citizens mobility (job offers). Besides, drug dealing and disappearances may be related to organized crime presence. We also observe a negative correlation between housing prices and perception of insecurity.

In Fig 3C we show the correlation matrix considering normalized disappearances and normalized reports of drug dealing. As we used population and job offers to normalize disappearances, we no longer included them in the correlation matrix. While Fig 3B shows that raw disappearances align with population mobility, Fig 3C reveals an independent socioeconomic gradient after controlling for exposure. A strong negative correlation emerges between housing prices and normalized disappearances, indicating that the economic level is involved in the probability of becoming a victim of disappearance. Housing prices are also negatively correlated with reports of drug dealing and perception of insecurity. Normalized disappearances are positively correlated with normalized reports of drug dealing and perception of insecurity.

These results corroborate the fact that Mexico City shows segregation, in which the level of economic standing is involved in the disappearance phenomenon and security in general.

### Clustering municipalities to identify the most vulnerable zones

To obtain a more general sense of the risk zones, and not only considering normalized disappearances, but also the economic and security environment variables, we cluster the municipalities considering the five variables: housing prices, normalized disappearances, normalized reports of drug dealing, perception of insecurity, and located individuals.

Previous to clustering municipalities, we implement dimension reduction to visualize in two dimensions the five variables. We implement principal component analysis (PCA) for dimensional reduction. PCA was used to retain the maximum variance in the five variables (5 dimensions). For example, a high variance is found between the maximum and minimum housing prices, which is approximately 6. In contrast, the ratio for located people is 1.3. The PCA assigns different values depending on the variable variation [39,40]

In Fig 4A we show the dimensional reduction for the 16 municipalities. The first principal component (PC1) accounts for 67.0% of the total variation, while the second (PC2) exhibits 13.62% of the total variation. We obtain the principal component, PC1=0.57(N.Dis.)−0.57(H.prices)−0.01(located)+0.34(N.drugd.)+0.47(p.ofIn.) The second component PC2=0.14(N.Dis.)−0.14(H.prices)−0.04(located)+0.58(N.drugd.) +0.78(p.of.In.). These components reduce all variables in two dimensions. It is important to note the inverse sign in PC loadings between normalized disappearances and housing prices, indicating a negative relation. Although the variance explained by the second component is small (13.62 %), it is necessary to retain more information from the original data and serves as a second attribute for implementing K-means clustering.

**Fig 4:**
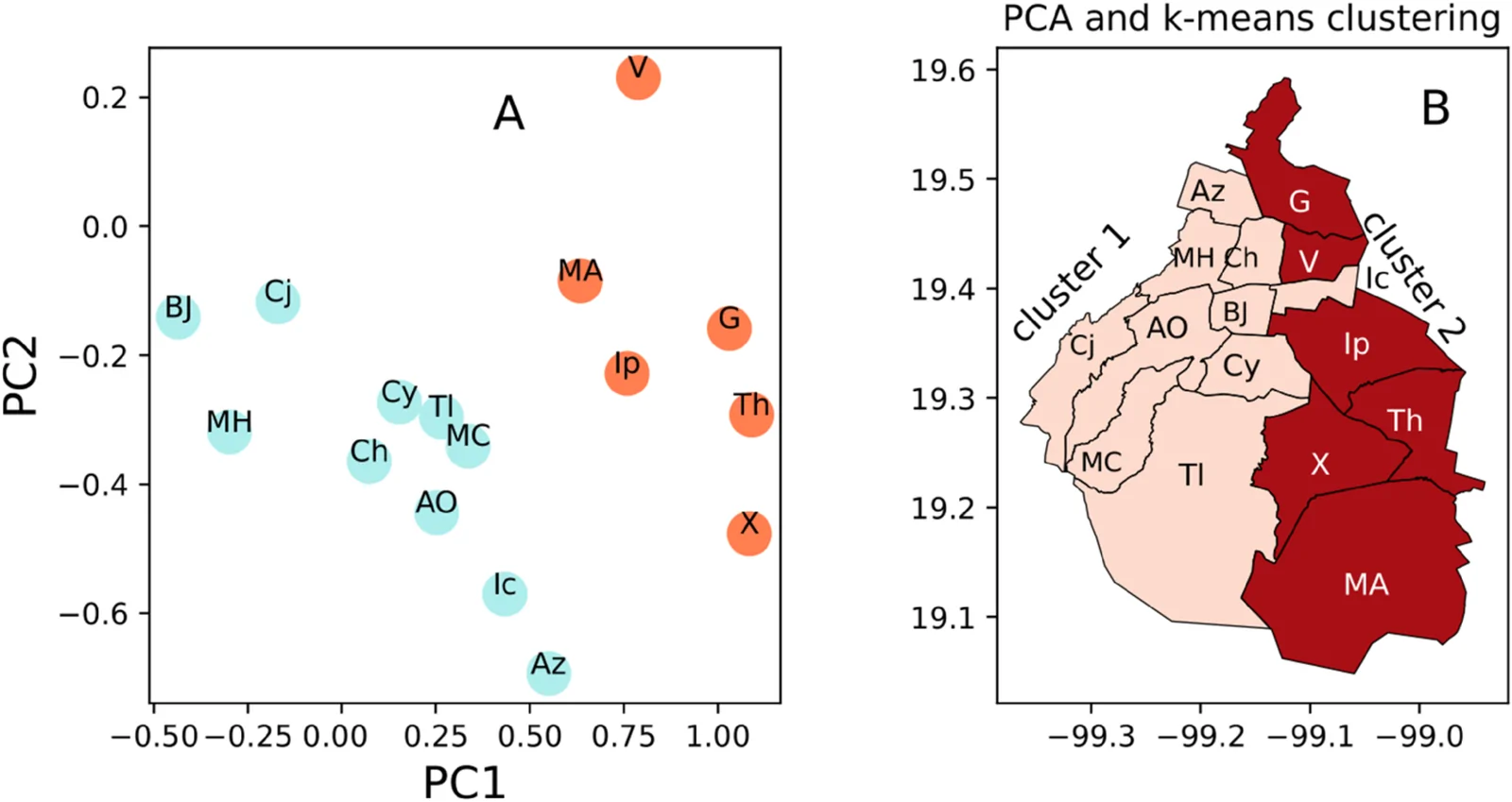
Dimensional reduction and clustering. A) Data was reduced from 5 to 2 dimensions (normalized disappearances, located/disappearances, housing prices, normalized reports of drug dealing, and security perception). Then, we applied K-means algorithm. Each cluster is represented by a different color. B) Mexico City map result from the K-means clustering.

Then, we apply the K-means algorithm to reveal the clustering of municipalities. We observe two main clusters. These two clusters are represented by different colors in the map of the Fig 4B. We observed that the city is divided into the west (cluster 1) and the east (cluster 2). It is worth to note that K-means does not use spatial data, so the east-west pattern arises only because variables cluster that way—not by geographic input. Cluster 1 is characterized by a high price for housing (Fig 2D), low to mid disappearances (Fig 3A) and a high number of people located (Fig 2C). Cluster 2 is characterized by middle to high disappearances (Fig 3A), low to middle located people (Fig 2C), and low housing prices (Fig 2D). These results support the hypothesis that high-income people can afford security, but only a small portion of the population can afford housing in the expensive zones and near their jobs [18].

## Conclusion

We analyzed the principal characteristics of missing people in Mexico City. In particular, we analyzed whether the disappearance phenomenon is linked to the socio-economic level. For women, the age of missing persons is mainly concentrated between 15-19 years, which may be related to human trafficking. This age interval may also be related to feminicides, caused primarily by partners, ex-partners, or acquaintances [44]. For men, the age of missing persons corresponds to the working age range of 15-44 years, which may be related to the recruitment of organized crime [7] and conflicts between drug cartels or dealers. The age distributions found for Mexico City are similar to the distributions found for the entire country [45], indicating an extended behavior.

For people who were located, the days that they were missing approximately follow a power-law function. This mathematical rule has been observed in economic and biological systems [46–48]. Most disappearances are concentrated in the central and eastern parts of the city, which can be attributed to the size and mobility of the population (Fig 2).

We tested the relationship between socioeconomic status and disappearances. To obtain this: (1) We propose a normalized version of disappearances that divides disappearances by population and mobility; (2) Housing prices are used as a measure of income range. After applying the above, a strong negative correlation emerges between economic income and disappearances (Fig 3B), indicating that wealthy individuals can access security more easily than the vast majority of people.

In contrast, not wealthy people commute for hours and are more susceptible to disappearing. This result also confirms the relation between crime and economic segregation observed in Mexico City [15,49]. In addition, the result agrees with a previous analysis considering disappearances before 2018, which found that the northeast of Mexico City is the most affected [50]. However, normalized disappearances indicate that the phenomenon extended to the southeast of Mexico City (see Fig 3).

Finally, integrating data from disappearances, security, and economic variables, K-means clustering divided Mexico City in the west and east. The most vulnerable are on the east side, close to the metropolitan zone of the state of Mexico, which has been very affected by organized crime. It is import to note that the K-means algorithm did not receive spatial information, however, segregation gives rise to this spatial pattern. This result corroborates the fact that machine learning can be useful in highlighting the most vulnerable people and, as a consequence, improve the quality of life of citizens [51].

We recommend the Mexican government to strengthen the personnel of the vigilance and justice system in the east of Mexico City and its metropolitan area; historically, both zones have been abandoned for justice, economic and labor opportunities. If impunity remains prevailing; the phenomenon may worsen in the east and will be extended to the west of the city.

## References

[1] Aureliani T. Desapariciones forzadas en América Latina y México: evolución del fenómeno y rol del crimen organizado. Foro internacional. 2023;63(4):730–47.

[2] Hernández A. Los señores del narco. Grijalbo; 2012.

[3] Tourliere M. México presume nuevo censo de desaparecidos ante críticas en la ONU por crisis de derechos humanos. 2024. https://www.proceso.com.mx/nacional/2024/1/24/mexico-presume-nuevo-censo-de-desaparecidos-ante-criticas-en-la-onu-por-crisis-de-derechos-humanos-html

[4] Molina H. Despliegue de fuerzas armadas, con poco impacto en las cifras de homicidios. El Economista. 2022.

[5] México E. México, el país que desaparece: sin rastro de 1250 00 personas. El País. 2023.

[6] Proceso. Los “abrazos, no balazos” de AMLO dejaron el sexenio más violento de México. Proceso. 2023.

[7] Prieto-Curiel R, Campedelli GM, Hope A. Reducing cartel recruitment is the only way to lower violence in Mexico. Science. 2023;381(6664):1312–6. doi: 10.1126/science.adh2888 37733856

[8] Arista L. La fragmentación de cárteles en México ha acentuado la violencia en 16 años. 2023. https://politica.expansion.mx/mexico/2023/03/01/fragmentacion-carteles-mexico-ha-acentuado-violencia

[9] NPR, Noroeste. Favorece guerra a cárteles de Sinaloa. 2015. https://www.noroeste.com.mx/nacional/favorece-guerra-a-cartel-de-sinaloa-JHNO242759

[10] El Paıs. Garcıa Luna: el castigo que México nunca impuso; 2024. https://elpais.com/mexico/opinion/2024-10-17/garcia-luna-el-castigo-que-mexico-nunca-impuso.html

[11] Hernández A. Militarización y trato preferencial al cártel de Sinaloa: las similitudes entre AMLO y Calderón según Anabel Hernández. 2022. https://www.infobae.com/america/mexico/2022/06/13/militarizacion-y-trato-preferencial-al-cartel-de-sinaloa-las-similitudes-entre-amlo-y-calderon-segun

[12] Coronado D, Rodríguez PH. Las desapariciones de personas: entre la degradación y la indiferencia. Universidad de Guadalajara. 2021.

[13] Davis DE. Disappearances in Mexico: Searching for Explanations Through Interdisciplinary Social Science; Lecture at Boston University, Pardee School of Global Studies. 2024.

[14] de Buśqueda CN. Versión Estadística RNPDNO. 2025. https://versionpublicarnpdno.segob.gob.mx/Dashboard/ContextoGeneral

[15] Saraví GA. Mundos aislados: segregación urbana y desigualdad en la ciudad de México. Eure. 2008;34(103):93–110.

[16] Aguilar AG, Ward PM, Smith Sr C. Globalization, regional development, and mega-city expansion in Latin America: analyzing Mexico City’s peri-urban hinterland. Cities. 2003;20(1):3–21.

[17] Székely M. La desigualdad en México: una perspectiva internacional. Washington: Banco Interamericano de Desarrollo. 1999.

[18] Aguilar-Velázquez D, Rivera Islas I, Romero Tecua G, Valenzuela-Aguilera A. Gentrification and access to housing in Mexico City during 2000 to 2022. Proc Natl Acad Sci U S A. 2024;121(10):e2314455121. doi: 10.1073/pnas.2314455121 38408232 PMC10927532

[19] Schatz S. Authorizing state crime in Mexico: the importance of a destructive social milieu. Crime, Law and Social Change. 2006;46:97–132.

[20] Vilalta C, Muggah R. What explains criminal violence in Mexico City? A test of two theories of crime. Stability: International Journal of Security and Development. 2016;5(1):1.

[21] Politico A. En CDMX, 98.4% de los delitos denunciados quedan impunes, narcomenudeo y desaparición llegan al 100% de impunidad. 2023. https://animalpolitico.com/seguridad/cdmx-delitos-denunciados-impunidad-mexico-evalua

[22] Raya-Tapia AY, Ramírez-Márquez C, Ponce-Ortega JM. Clustering of zones according to the level of gentrification by using an unsupervised learning algorithm. Cities. 2024;151:105133.

[23] Cohen LE, Felson M. Social change and crime rate trends: A routine activity approach. Classics in environmental criminology. Routledge; 2010. p. 203–32.

[24] Miró F. Routine activity theory. The encyclopedia of theoretical criminology. 2014. p. 1–7.

[25] Brantingham P, Brantingham P. Crime pattern theory. Environmental criminology and crime analysis. Willan; 2013. p. 100–16.

[26] Navarro MF. Población flotante: DF, centro laboral y escolar del Edomex. 2015. https://www.excelsior.com.mx/comunidad/2015/12/24/1065140

[27] Simancas JV. As ı se escapa la vida de Adriana: cinco horas de transporte pu´blico al d´ıa para poder trabajar. 2024. https://elpais.com/mexico/2024-01-28/asi-se-escapa-la-vida-de-adriana-cinco-horas-de-transporte-publico-al-dia-para-poder-trabajar.html

[28] Piña-García CA, Ramírez-Ramírez L. Exploring crime patterns in Mexico City. Journal of Big Data. 2019;6:1–21.

[29] Valenzuela-Aguilera A. The spatial dimension of crime in Mexico City 2016 –2019). Rice University’s Baker Institute for Public Policy;; 2020.

[30] Gob-CDMX. Version publica del registro interno de personas desaparecida. 2023. https://comisiondebusqueda.cdmx.gob.mx/CBP/rpd/version-publica

[31] Valenzuela-Aguilera A, Romero GT. La ciudad de los 15 minutos y la escala metropolitana: l caso de la Zona Metropolitana del Valle de México. Ciudad y territorio: Estudios territoriales. 2024;220:391–402.

[32] SNE. Diagnóstico del mercado laboral de la ciudad de México. 2024. https://www.observatoriolaboral.gob.mx/static/estudios-publicaciones/CDMX.pdf

[33] INEGI. Numero de habitantes. 2020. https://cuentame.inegi.org.mx/monografias/informacion/df/poblacion/

[34] Pangallo M, Nadal JP, Vignes A. Residential income segregation: a behavioral model of the housing market. Journal of Economic Behavior & Organization. 2019;159:15–35.

[35] Aguilera A, Ugalde E. A spatially extended model for residential segregation. Discrete Dynamics in Nature and Society. 2007;2007(1):048589.

[36] Zerega G. Mexico City’s lucrative real estate scams. El País. 2023.

[37] Gobierno C. Datos abiertos de incidencia delictiva. 2024. https://www.gob.mx/sesnsp/acciones-y-programas/datos-abiertos-de-incidencia-delictiva?state=published

[38] INEGI. Encuesta nacional de seguridad pública urbana. 2024. https://www.inegi.org.mx/contenidos/saladeprensa/boletines/2024/ENSU/ENSU2024_07.pdf.

[39] Ringnér M. What is principal component analysis?. Nat Biotechnol. 2008;26(3):303–4. doi: 10.1038/nbt0308-303 18327243

[40] Jolliffe IT, Cadima J. Principal component analysis: a review and recent developments. Philos Trans A Math Phys Eng Sci. 2016;374(2065):20150202. doi: 10.1098/rsta.2015.0202 26953178 PMC4792409

[41] Hartigan JA, Wong MA. A k-means clustering algorithm. Applied Statistics. 1979;28(1):100–8.

[42] Thorndike RL. Who belongs in the family?. Psychometrika. 1953;18(4):267–76.

[43] Hanel R, Corominas-Murtra B, Liu B, Thurner S. Fitting power-laws in empirical data with estimators that work for all exponents. PLoS One. 2017;12(2):e0170920. doi: 10.1371/journal.pone.0170920 28245249 PMC5330461

[44] Yardley E. The Killing of Women in “Sex Games Gone Wrong”: An Analysis of Femicides in Great Britain 2000 -2018. Violence Against Women. 2021;27(11):1840–61. doi: 10.1177/1077801220966956 33143567

[45] IMDHD. Informe en línea. [cited 2024 May 1]. https://imdhd.org/redlupa/wp-content/uploads/2024/05/Informe-Nacional-2024.pdf

[46] Newman ME. Power laws, Pareto distributions and Zipf’s law. Contemporary Physics. 2005;46(5):323–51.

[47] Aguilar-Velázquez D, Guzmán-Vargas L. Critical synchronization and 1/f noise in inhibitory/excitatory rich-club neural networks. Sci Rep. 2019;9(1):1258. doi: 10.1038/s41598-018-37920-w 30718817 PMC6361933

[48] Aguilar-Velazquez D. Critical neural networks minimize metabolic cost. Physics. 2021;3(1):42–58.

[49] Capron G. Seguridad, desconfianza y la dimensioń simbólica de la segregación en urbanizaciones cerradas. EURE. 2021;47(142):121–37.

[50] Chavez LGB. Del análisis espacial a una aproximación teórica hacia los espacios de ausencia a partir de la desaparición involuntaria de personas en la ZMVM. Unknown Journal. 2023.

[51] Valenzuela-Aguilera A, Romero-Tecua G, Aguilar-Velázquez D, Islas IR. Smart cities: aligning tech efficiency with social justice. SN Computer Science. 2024;5(8):997.

