# Supplementary material for: Understanding disappearances in Mexico City: A data-driven analysis
Source: PLoS One. 2025 Sep 23;20(9):e0323405. doi: 10.1371/journal.pone.0323405 (PMC13652337; doi:10.1371/journal.pone.0323405)
Supplement: Supplementary file 1 [file pone.0323405.s001.docx]

Reviewer #1

First, the introduction should be expanded. In particular, the authors greatly simplify two criminological theories – social disorganization and anomie. I suggest that the authors devote more time to discussing these theoretical perspectives. Relatedly, why did the authors choose these two theories to discuss? There are several theories that link place to crime. More justification for why the authors discuss these theories is warranted.

We indicate that several theories are required to explain the phenomenon of disappearances (lines 19–21). In addition, we explain why these two theories, along with the criminogenic state theory, better fit the case of Mexico City (lines 25–32)

Also in the introduction, the authors discuss crime – conceptually and theoretically – instead of missing persons. These are not synonymous. Rather, the authors should discuss missing persons in more depth. What theories explain this phenomenon? How many missing persons are there annually? Is there an upward trend in missing persons? What do we already know about missing persons? What will this study add beyond what we already know about missing persons?

We expanded the introduction explaining why there was a dramatic increase in disappearances since 2006, showing a persistent upward trend, and pointing organized crime as a central disappearances perpetrator (lines 2-13).

The authors claim that they will investigate the relationship between disappearances and economic inequality. However, they do not have measures of economic inequality. Rather, they examine housing prices, an indicator of socioeconomic status (not inequality). Please characterize this correctly.

The economic inequality concept was removed, and we used “socioeconomic status” instead. We also used the term “income range” as recommended by the Reviewer #2

The analyses were well-done, and the results are very interesting. However, some of the figures were overwhelming. For example, there are 16 maps presented in figure 2, even though there was very little variation across the total, male, and female maps. The authors should streamline the results and tables/figures presented so as to highlight the key findings.

We modified Figure 2, which now displays eight maps, avoiding redundancy between adult and overall disappearances, as well as redundancy related to located individuals. We also revised the text to align with the updated figure.

Additionally, the conclusion section simply summarizes the results that were just presented. There is very little interpretation of the findings or discussion of policy recommendations to limit missing persons and increase the likelihood that missing persons are found

We included a discussion comparing our results with previous findings (lines 276–277 and 291–294). We also added policy recommendations (lines 302–306), mainly focused on strengthening the security and justice systems in the eastern part of Mexico City.

Reviewer #2:

Introduction From line 20 on in the introduction – this is a mixture of introduction content and methodology. Please move almost all of this content to the methods section.

We moved the paragraph: *To obtain a more precise measure of disappearances...* (previously in line 20) to the methods section (now in lines 56-59 and lines 72-75)

Materials and methods Does housing price proxy income inequality well? I am not so sure it does. I think you might be better off using income range (highest-lowest), which captures absolute difference within a given municipality between low and high, or a housing price range – in any case, for inequality, I think you would be better off using a range. If not, please explain why or at the very least provide more information (in this section, not the introduction) as to why this is measure is appropriate and gets at what you are trying to proxy.

The “economic inequality” concept was removed and we used “income range” instead. We also used “socioeconomic status” as recommended by the Reviewer #1

I think you could benefit from a line explaining the geometric mean – for readers unfamiliar, it can helpful to explain why this specific averaging approach is better than arithmetic mean or regression based adjustment

A brief explanation of the geometric mean, and why it is preferable to the arithmetic mean when using different data scales, is provided in lines 62–66

Although you noted this later, I think there should be more discussion in the methods about the limitations on being found (e.g., no information on mortality), which is a major limitation in the inferences you can make

We include the following lines (129-131): The database does not specify whether the people located are dead or alive. This represents a problem because we cannot infer if the location of people is a positive or negative parameter.

Results (disappearances by gender, age, and year)

Are these values relative to total populations? If not, then the estimates are materially biased (e.g., if there are more men than women then the finding that men disappear more frequently is biased). It seems like they must be, since authors have done a good job standardizing many other variables, but that is not mentioned here to my knowledge

The values are relative to total populations indicated in lines 152 and 153. We also mention the percentage of men and women population in Mexico City.

You mention P(days) = 0.14 x days^-0.4, which is plausible for a power-law. But how was this fitted? Was it visually assessed, or was a specific estimator (e.g., maximum likelihood) used?

In the previous version, we used the least mean squares algorithm on a log-log scale (which was not mentioned in the manuscript). However, as you pointed out, maximum likelihood (ML) is a more appropriate method. We implemented the ML* [1] method to estimate the power-law exponent. After applying this method, we found α = 0.36 ± 0.11, where ± indicates the mean square error of the power-law fit (lines 162–165)

Fig 3A interpretation could be more precise. When saying “disappearances are higher in the east,” make sure to emphasize that this is per normalized exposure—i.e., after adjusting for both size and commuting density.

We emphasize that this is per normalized exposure in (lines 205-206)

You move from raw to normalized values. This is great, but a sentence contrasting the difference in interpretation between 3B and 3C would help. For example, “While 3B shows that raw disappearances align with population mobility, 3C reveals an independent socioeconomic gradient after controlling for exposure.”

We introduce the suggested sentence in lines 223-225

The clustering output is well-presented, but the conclusion that “the city divides into east and west” could be presented with a cautionary note: K-means does not use spatial data, so the east-west pattern arises only because variables cluster that way—not by geographic input.

We introduce the sentence in lines 260-262

Sometimes “people located” and “people found” are used interchangeably, but remember: the dataset doesn’t specify whether they were alive. Be consistent and careful—use “located” unless you are certain.

In the revised manuscript, we use only the term “located people”

The second principal component (PC2) appears to explain little variance and may not contribute meaningful differentiation between municipalities. Please clarify its role and justify the use of PCA with two components.

We added the following lines (254–256): Although the variance explained by the second component is small (13.62%), it is necessary to retain more information from the original data and serves as a second attribute for implementing K-means clustering.

Conclusion The statement that “victims exhibit certain profiles that make them ideal targets for criminals” overreaches the evidence presented. The data support associations, but there is no direct evidence of targeting mechanisms. The authors should revise this language to avoid implying intent or causality where it is not demonstrated.

We removed the mentioned statement (previously in the first paragraph of the conclusion) and revised the language to avoid implying causality that is not supported by the results.

The conclusion references the power-law distribution of disappearance durations. While the fit appears plausible, the authors should describe it more cautiously, noting that the distribution “approximates” a power-law and that the fit was based on empirical observation rather than formal estimation procedures

We implement ML* method to estimate the power-law exponent as indicated previously. In addition, we include the word “approximates” in the conclusion (line 278) due to the mean square error presented in the results.

Reference

[1] Hanel R, Corominas-Murtra B, Liu B, Thurner S. Fitting power-laws in empirical data with estimators that work for all exponents. PloS one. 2017;12(2):e0170920
