# Supplementary material for: Understanding disappearances in Mexico City: A data-driven analysis
Source: PLoS One. 2025 Sep 23;20(9):e0323405. doi: 10.1371/journal.pone.0323405 (PMC13652337; doi:10.1371/journal.pone.0323405)
Supplement: Peer review history

# Peer review: Decision Letter 0

**DOI:** 10.1371/journal.pone.0323405.r001

**Authors:** Claudio Dávila-Cervantes

15 May 2025

PONE-D-25-14641Understanding disappearances in Mexico City: a data-driven analysisPLOS ONE

Dear Dr. Aguilar-Velazquez,

Thank you for submitting your manuscript to PLOS ONE. After careful consideration, we feel that it has merit but does not fully meet PLOS ONE’s publication criteria as it currently stands. Therefore, we invite you to submit a revised version of the manuscript that addresses the points raised during the review process.

**ACADEMIC EDITOR:**

Thank you for submitting your manuscript. The peer-review process has been completed. The reviewers have requested, a few minor issues need to be addressed before the manuscript can be considered for publication. The detailed feedback from reviewers is included below/attached for your reference.

We kindly request that you address these points in your revised manuscript and provide a response letter detailing the changes made. Please submit the revised version of your manuscript along with the response letter through our submission system.

If you have any questions or require clarification regarding the reviewers' comments, please feel free to contact us. We look forward to receiving your revised manuscript.

Please submit your revised manuscript by Jun 29 2025 11:59PM. If you will need more time than this to complete your revisions, please reply to this message or contact the journal office at plosone@plos.org. When you're ready to submit your revision, log on to https://www.editorialmanager.com/pone/ and select the 'Submissions Needing Revision' folder to locate your manuscript file.

Please include the following items when submitting your revised manuscript:

- A rebuttal letter that responds to each point raised by the academic editor and reviewer(s). You should upload this letter as a separate file labeled 'Response to Reviewers'.
- A marked-up copy of your manuscript that highlights changes made to the original version. You should upload this as a separate file labeled 'Revised Manuscript with Track Changes'.
- An unmarked version of your revised paper without tracked changes. You should upload this as a separate file labeled 'Manuscript'.

If you would like to make changes to your financial disclosure, please include your updated statement in your cover letter. Guidelines for resubmitting your figure files are available below the reviewer comments at the end of this letter.

If applicable, we recommend that you deposit your laboratory protocols in protocols.io to enhance the reproducibility of your results. Protocols.io assigns your protocol its own identifier (DOI) so that it can be cited independently in the future. For instructions see: https://journals.plos.org/plosone/s/submission-guidelines#loc-laboratory-protocols. Additionally, PLOS ONE offers an option for publishing peer-reviewed Lab Protocol articles, which describe protocols hosted on protocols.io. Read more information on sharing protocols at https://plos.org/protocols?utm_medium=editorial-email&utm_source=authorletters&utm_campaign=protocols.

We look forward to receiving your revised manuscript.

Kind regards,

Claudio Alberto Dávila-Cervantes, Ph.D.

Academic Editor

PLOS ONE

Journal Requirements:

When submitting your revision, we need you to address these additional requirements.

1. Please ensure that your manuscript meets PLOS ONE's style requirements, including those for file naming. The PLOS ONE style templates can be found at

https://journals.plos.org/plosone/s/file?id=wjVg/PLOSOne_formatting_sample_main_body.pdf and

https://journals.plos.org/plosone/s/file?id=ba62/PLOSOne_formatting_sample_title_authors_affiliations.pdf

2. Please note that PLOS ONE has specific guidelines on code sharing for submissions in which author-generated code underpins the findings in the manuscript. In these cases, we expect all author-generated code to be made available without restrictions upon publication of the work. Please review our guidelines at https://journals.plos.org/plosone/s/materials-and-software-sharing#loc-sharing-code and ensure that your code is shared in a way that follows best practice and facilitates reproducibility and reuse.

3. We note that the grant information you provided in the ‘Funding Information’ and ‘Financial Disclosure’ sections do not match.

When you resubmit, please ensure that you provide the correct grant numbers for the awards you received for your study in the ‘Funding Information’ section.

4. Thank you for stating the following in the Acknowledgments Section of your manuscript: [Daniel Aguilar-Vel´azquez thanks CONAHCYT for their support through grant 481209.]

We note that you have provided funding information that is not currently declared in your Funding Statement. However, funding information should not appear in the Acknowledgments section or other areas of your manuscript. We will only publish funding information present in the Funding Statement section of the online submission form.

Please remove any funding-related text from the manuscript and let us know how you would like to update your Funding Statement. Currently, your Funding Statement reads as follows: “The authors received no specific funding for this work”.

Please include your amended statements within your cover letter; we will change the online submission form on your behalf.

5. Please review your reference list to ensure that it is complete and correct. If you have cited papers that have been retracted, please include the rationale for doing so in the manuscript text, or remove these references and replace them with relevant current references. Any changes to the reference list should be mentioned in the rebuttal letter that accompanies your revised manuscript. If you need to cite a retracted article, indicate the article’s retracted status in the References list and also include a citation and full reference for the retraction notice.

[Note: HTML markup is below. Please do not edit.]

Reviewers' comments:

Reviewer's Responses to Questions

**Comments to the Author**

1. Is the manuscript technically sound, and do the data support the conclusions?

The manuscript must describe a technically sound piece of scientific research with data that supports the conclusions. Experiments must have been conducted rigorously, with appropriate controls, replication, and sample sizes. The conclusions must be drawn appropriately based on the data presented. 

Reviewer #1: Partly

Reviewer #2: Partly

**********

2. Has the statistical analysis been performed appropriately and rigorously? 

Reviewer #1: Yes

Reviewer #2: Yes

**********

3. Have the authors made all data underlying the findings in their manuscript fully available?

The PLOS Data policy requires authors to make all data underlying the findings described in their manuscript fully available without restriction, with rare exception (please refer to the Data Availability Statement in the manuscript PDF file). The data should be provided as part of the manuscript or its supporting information, or deposited to a public repository. For example, in addition to summary statistics, the data points behind means, medians and variance measures should be available. If there are restrictions on publicly sharing data—e.g. participant privacy or use of data from a third party—those must be specified.

Reviewer #1: Yes

Reviewer #2: Yes

**********

4. Is the manuscript presented in an intelligible fashion and written in standard English?

PLOS ONE does not copyedit accepted manuscripts, so the language in submitted articles must be clear, correct, and unambiguous. Any typographical or grammatical errors should be corrected at revision, so please note any specific errors here.

Reviewer #1: Yes

Reviewer #2: Yes

**********

5. Review Comments to the Author

Please use the space provided to explain your answers to the questions above. You may also include additional comments for the author, including concerns about dual publication, research ethics, or publication ethics. (Please upload your review as an attachment if it exceeds 20,000 characters)

Reviewer #1: I appreciate the opportunity to review manuscript PONE-D-25-14641 entitled “Understanding disappearances in Mexico City: a data-driven analysis.” The topic is compelling, and I appreciate the authors’ data-driven approach to understanding missing persons. I have several suggestions that could improve the manuscript as is currently written.

First, the introduction should be expanded. In particular, the authors greatly simplify two criminological theories – social disorganization and anomie. I suggest that the authors devote more time to discussing these theoretical perspectives. Relatedly, why did the authors choose these two theories to discuss? There are several theories that link place to crime. More justification for why the authors discuss these theories is warranted.

Also in the introduction, the authors discuss crime – conceptually and theoretically – instead of missing persons. These are not synonymous. Rather, the authors should discuss missing persons in more depth. What theories explain this phenomenon? How many missing persons are there annually? Is there an upward trend in missing persons? What do we already know about missing persons? What will this study add beyond what we already know about missing persons?

The authors claim that they will investigate the relationship between disappearances and economic inequality. However, they do not have measures of economic inequality. Rather, they examine housing prices, an indicator of socioeconomic status (not inequality). Please characterize this correctly.

The analyses were well-done, and the results are very interesting. However, some of the figures were overwhelming. For example, there are 16 maps presented in figure 2, even though there was very little variation across the total, male, and female maps. The authors should streamline the results and tables/figures presented so as to highlight the key findings.

Additionally, the conclusion section simply summarizes the results that were just presented. There is very little interpretation of the findings or discussion of policy recommendations to limit missing persons and increase the likelihood that missing persons are found.

Reviewer #2: Introduction

From line 20 on in the introduction – this is a mixture of introduction content and methodology. Please move almost all of this content to the methods section.

Materials and methods

Does housing price proxy income inequality well? I am not so sure it does. I think you might be better off using income range (highest-lowest), which captures absolute difference within a given municipality between low and high, or a housing price range – in any case, for inequality, I think you would be better off using a range. If not, please explain why or at the very least provide more information (in this section, not the introduction) as to why this is measure is appropriate and gets at what you are trying to proxy.

I think you could benefit from a line explaining the geometric mean – for readers unfamiliar, it can helpful to explain why this specific averaging approach is better than arithmetic mean or regression based adjustment

Although you noted this later, I think there should be more discussion in the methods about the limitations on being found (e.g., no information on mortality), which is a major limitation in the inferences you can make

Results (disappearances by gender, age, and year)

Are these values relative to total populations? If not, then the estimates are materially biased (e.g., if there are more men than women then the finding that men disappear more frequently is biased). It seems like they must be, since authors have done a good job standardizing many other variables, but that is not mentioned here to my knowledge

You mention P(days) = 0.14 x days^-0.4, which is plausible for a power-law. But how was this fitted? Was it visually assessed, or was a specific estimator (e.g., maximum likelihood) used?

Fig 3A interpretation could be more precise. When saying “disappearances are higher in the east,” make sure to emphasize that this is per normalized exposure—i.e., after adjusting for both size and commuting density.

You move from raw to normalized values. This is great, but a sentence contrasting the difference in interpretation between 3B and 3C would help. For example, “While 3B shows that raw disappearances align with population mobility, 3C reveals an independent socioeconomic gradient after controlling for exposure.”

The clustering output is well-presented, but the conclusion that “the city divides into east and west” could be presented with a cautionary note: K-means does not use spatial data, so the east-west pattern arises only because variables cluster that way—not by geographic input.

Sometimes “people located” and “people found” are used interchangeably, but remember: the dataset doesn’t specify whether they were alive. Be consistent and careful—use “located” unless you are certain.

The second principal component (PC2) appears to explain little variance and may not contribute meaningful differentiation between municipalities. Please clarify its role and justify the use of PCA with two components.

Conclusion

The statement that “victims exhibit certain profiles that make them ideal targets for criminals” overreaches the evidence presented. The data support associations, but there is no direct evidence of targeting mechanisms. The authors should revise this language to avoid implying intent or causality where it is not demonstrated.

The conclusion references the power-law distribution of disappearance durations. While the fit appears plausible, the authors should describe it more cautiously, noting that the distribution “approximates” a power-law and that the fit was based on empirical observation rather than formal estimation procedures.

Overall, I find this to be a fascinating and transparently conducted study with compelling descriptive findings. However, I strongly encourage the authors to temper their causal language throughout and instead focus on the two areas where the analysis is most robust and defensible: (1) the demographic patterns in disappearances—particularly the age differences between men and women—and (2) the geographic distribution of disappearance risk across municipalities. Attempts to draw inferences about causes or associated factors go beyond what the current data and methods support. The models, while innovative in structure, are too sparse and were not designed to answer causal questions.

**********

6. PLOS authors have the option to publish the peer review history of their article (what does this mean?). If published, this will include your full peer review and any attached files.

If you choose “no”, your identity will remain anonymous but your review may still be made public.

**Do you want your identity to be public for this peer review?** For information about this choice, including consent withdrawal, please see our Privacy Policy.

Reviewer #1: No

Reviewer #2: No

**********

[NOTE: If reviewer comments were submitted as an attachment file, they will be attached to this email and accessible via the submission site. Please log into your account, locate the manuscript record, and check for the action link "View Attachments". If this link does not appear, there are no attachment files.]

While revising your submission, please upload your figure files to the Preflight Analysis and Conversion Engine (PACE) digital diagnostic tool, https://pacev2.apexcovantage.com/. PACE helps ensure that figures meet PLOS requirements. To use PACE, you must first register as a user. Registration is free. Then, login and navigate to the UPLOAD tab, where you will find detailed instructions on how to use the tool. If you encounter any issues or have any questions when using PACE, please email PLOS at figures@plos.org. Please note that Supporting Information files do not need this step.

# Author comment: Author response to Decision Letter 1

**DOI:** 10.1371/journal.pone.0323405.r002

18 Jul 2025

Reviewer #1

First, the introduction should be expanded. In particular, the authors greatly simplify two criminological theories – social disorganization and anomie. I suggest that the authors devote more time to discussing these theoretical perspectives. Relatedly, why did the authors choose these two theories to discuss? There are several theories that link place to crime. More justification for why the authors discuss these theories is warranted.

-We indicate that several theories are required to explain the phenomenon of disappearances (lines 19–21). In addition, we explain why these two theories, along with the criminogenic state theory, better fit the case of Mexico City (lines 25–32)

Also in the introduction, the authors discuss crime – conceptually and theoretically – instead of missing persons. These are not synonymous. Rather, the authors should discuss missing persons in more depth. What theories explain this phenomenon? How many missing persons are there annually? Is there an upward trend in missing persons? What do we already know about missing persons? What will this study add beyond what we already know about missing persons?

-We expanded the introduction explaining why there was a dramatic increase in disappearances since 2006, showing a persistent upward trend, and pointing organized crime as a central disappearances perpetrator (lines 2-13).

The authors claim that they will investigate the relationship between disappearances and economic inequality. However, they do not have measures of economic inequality. Rather, they examine housing prices, an indicator of socioeconomic status (not inequality). Please characterize this correctly.

-The economic inequality concept was removed, and we used “socioeconomic status” instead. We also used the term “income range” as recommended by the Reviewer #2

The analyses were well-done, and the results are very interesting. However, some of the figures were overwhelming. For example, there are 16 maps presented in figure 2, even though there was very little variation across the total, male, and female maps. The authors should streamline the results and tables/figures presented so as to highlight the key findings.

-We modified Figure 2, which now displays eight maps, avoiding redundancy between adult and overall disappearances, as well as redundancy related to located individuals. We also revised the text to align with the updated figure.

Additionally, the conclusion section simply summarizes the results that were just presented. There is very little interpretation of the findings or discussion of policy recommendations to limit missing persons and increase the likelihood that missing persons are found

We included a discussion comparing our results with previous findings (lines 276–277 and 291–294).

- We also added policy recommendations (lines 302–306), mainly focused on strengthening the security and justice systems in the eastern part of Mexico City.

Reviewer #2:

Introduction From line 20 on in the introduction – this is a mixture of introduction content and methodology. Please move almost all of this content to the methods section.

-We moved the paragraph: To obtain a more precise measure of disappearances... (previously in line 20) to the methods section (now in lines 56-59 and lines 72-75)

Materials and methods Does housing price proxy income inequality well? I am not so sure it does. I think you might be better off using income range (highest-lowest), which captures absolute difference within a given municipality between low and high, or a housing price range – in any case, for inequality, I think you would be better off using a range. If not, please explain why or at the very least provide more information (in this section, not the introduction) as to why this is measure is appropriate and gets at what you are trying to proxy.

-The “economic inequality” concept was removed and we used “income range” instead. We also used “socioeconomic status” as recommended by the Reviewer #1

I think you could benefit from a line explaining the geometric mean – for readers unfamiliar, it can helpful to explain why this specific averaging approach is better than arithmetic mean or regression based adjustment

-A brief explanation of the geometric mean, and why it is preferable to the arithmetic mean when using different data scales, is provided in lines 62–66

Although you noted this later, I think there should be more discussion in the methods about the limitations on being found (e.g., no information on mortality), which is a major limitation in the inferences you can make

-We include the following lines (129-131): The database does not specify whether the people located are dead or alive. This represents a problem because we cannot infer if the

location of people is a positive or negative parameter.

Results (disappearances by gender, age, and year)

Are these values relative to total populations? If not, then the estimates are materially biased (e.g., if there are more men than women then the finding that men disappear more frequently is biased). It seems like they must be, since authors have done a good job standardizing many other variables, but that is not mentioned here to my knowledge

-The values are relative to total populations indicated in lines 152 and 153. We also mention the percentage of men and women population in Mexico City.

You mention P(days) = 0.14 x days^-0.4, which is plausible for a power-law. But how was this fitted? Was it visually assessed, or was a specific estimator (e.g., maximum likelihood) used?

-In the previous version, we used the least mean squares algorithm on a log-log scale (which was not mentioned in the manuscript). However, as you pointed out, maximum likelihood (ML) is a more appropriate method. We implemented the ML* [1] method to estimate the power-law exponent. After applying this method, we found α = 0.36 ± 0.11, where ± indicates the mean square error of the power-law fit (lines 162–165)

Fig 3A interpretation could be more precise. When saying “disappearances are higher in the east,” make sure to emphasize that this is per normalized exposure—i.e., after adjusting for both size and commuting density.

-We emphasize that this is per normalized exposure in (lines 205-206)

You move from raw to normalized values. This is great, but a sentence contrasting the difference in interpretation between 3B and 3C would help. For example, “While 3B shows that raw disappearances align with population mobility, 3C reveals an independent socioeconomic gradient after controlling for exposure.”

-We introduce the suggested sentence in lines 223-225

The clustering output is well-presented, but the conclusion that “the city divides into east and west” could be presented with a cautionary note: K-means does not use spatial data, so the east-west pattern arises only because variables cluster that way—not by geographic input.

-We introduce the sentence in lines 260-262

Sometimes “people located” and “people found” are used interchangeably, but remember: the dataset doesn’t specify whether they were alive. Be consistent and careful—use “located” unless you are certain.

-In the revised manuscript, we use only the term “located people”

The second principal component (PC2) appears to explain little variance and may not contribute meaningful differentiation between municipalities. Please clarify its role and justify the use of PCA with two components.

-We added the following lines (254–256): Although the variance explained by the second component is small (13.62%), it is necessary to retain more information from the original data and serves as a second attribute for implementing K-means clustering.

Conclusion The statement that “victims exhibit certain profiles that make them ideal targets for criminals” overreaches the evidence presented. The data support associations, but there is no direct evidence of targeting mechanisms. The authors should revise this language to avoid implying intent or causality where it is not demonstrated.

-We removed the mentioned statement (previously in the first paragraph of the conclusion) and revised the language to avoid implying causality that is not supported by the results.

The conclusion references the power-law distribution of disappearance durations. While the fit appears plausible, the authors should describe it more cautiously, noting that the distribution “approximates” a power-law and that the fit was based on empirical observation rather than formal estimation procedures

-We implement ML* method to estimate the power-law exponent as indicated previously. In addition, we include the word “approximates” in the conclusion (line 278) due to the mean square error presented in the results.

Reference

[1] Hanel R, Corominas-Murtra B, Liu B, Thurner S. Fitting power-laws in empirical data with estimators that work for all exponents. PloS one. 2017;12(2):e0170920

Attachment Submitted filename: response to reviewers.docx

# Editor report: Decision Letter 1

**DOI:** 10.1371/journal.pone.0323405.r003

**Authors:** Claudio Dávila-Cervantes

22 Jul 2025

Understanding disappearances in Mexico City: a data-driven analysis

PONE-D-25-14641R1

Dear Dr. Aguilar-Velazquez,

We’re pleased to inform you that your manuscript has been judged scientifically suitable for publication and will be formally accepted for publication once it meets all outstanding technical requirements.

Within one week, you’ll receive an e-mail detailing the required amendments. When these have been addressed, you’ll receive a formal acceptance letter and your manuscript will be scheduled for publication.

An invoice will be generated when your article is formally accepted. Please note, if your institution has a publishing partnership with PLOS and your article meets the relevant criteria, all or part of your publication costs will be covered. Please make sure your user information is up-to-date by logging into Editorial Manager at Editorial Manager® and clicking the ‘Update My Information' link at the top of the page. If you have any questions relating to publication charges, please contact our Author Billing department directly at authorbilling@plos.org.

If your institution or institutions have a press office, please notify them about your upcoming paper to help maximize its impact. If they’ll be preparing press materials, please inform our press team as soon as possible -- no later than 48 hours after receiving the formal acceptance. Your manuscript will remain under strict press embargo until 2 pm Eastern Time on the date of publication. For more information, please contact onepress@plos.org.

Kind regards,

Claudio Alberto Dávila-Cervantes, Ph.D.

Academic Editor

PLOS ONE

Additional Editor Comments (optional):

Reviewers' comments:

# Editor report: Acceptance letter

**DOI:** 10.1371/journal.pone.0323405.r004

**Authors:** Claudio Dávila-Cervantes

PONE-D-25-14641R1

PLOS ONE

Dear Dr. Aguilar-Velazquez,

I'm pleased to inform you that your manuscript has been deemed suitable for publication in PLOS ONE. Congratulations! Your manuscript is now being handed over to our production team.

At this stage, our production department will prepare your paper for publication. This includes ensuring the following:

* All references, tables, and figures are properly cited

* All relevant supporting information is included in the manuscript submission,

* There are no issues that prevent the paper from being properly typeset

You will receive further instructions from the production team, including instructions on how to review your proof when it is ready. Please keep in mind that we are working through a large volume of accepted articles, so please give us a few days to review your paper and let you know the next and final steps.

Lastly, if your institution or institutions have a press office, please let them know about your upcoming paper now to help maximize its impact. If they'll be preparing press materials, please inform our press team within the next 48 hours. Your manuscript will remain under strict press embargo until 2 pm Eastern Time on the date of publication. For more information, please contact onepress@plos.org.

You will receive an invoice from PLOS for your publication fee after your manuscript has reached the completed accept phase. If you receive an email requesting payment before acceptance or for any other service, this may be a phishing scheme. Learn how to identify phishing emails and protect your accounts at https://explore.plos.org/phishing.

If we can help with anything else, please email us at customercare@plos.org.

Thank you for submitting your work to PLOS ONE and supporting open access.

Kind regards,

PLOS ONE Editorial Office Staff

on behalf of

Mr. Claudio Alberto Dávila-Cervantes

Academic Editor

PLOS ONE
